# Lower Life Satisfaction and Inflammation in African American Adults: Body Adiposity Mediation and Sex Moderation

**DOI:** 10.3390/jpm12050745

**Published:** 2022-05-04

**Authors:** Kandauda A. S. Wickrama, Penny A. Ralston, Jasminka Z. Ilich

**Affiliations:** 1 Department of Human Development and Family Science, University of Georgia, Athens, GA 30602, USA; wickrama@uga.edu; 2 Center on Better Health and Life for Underserved Populations, Florida State University, Tallahassee, FL 32306, USA; pralston@fsu.edu

**Keywords:** life satisfaction, stress, inflammation, C-reactive protein, African Americans, body adiposity, BMI

## Abstract

Both lower life satisfaction (LLS) and chronic inflammation are underlying conditions for numerous diseases. We investigated their associations in African American adults, within the context of three hypotheses: (a) perceived LLS will be positively associated with inflammation measured by serum C-reactive protein (CRP); (b) this association will be mediated by body adiposity; and (c) these associations will be moderated by sex. Participants (*n* = 83; >45 years; 59% women) were a subsample of a larger church-based intervention to reduce cardiovascular risks and were assessed at baseline and after 6 months. Body adiposity (BMI/hip/waist circumferences) was measured by standardized methods and CRP with ELISA. LLS was self-reported. The analyses were conducted in the structural equation modeling (SEM) framework. The direct relationship between LLS and CRP was significant for all participants but was mediated by BMI/hip/waist circumferences. Multi-group SEM analysis provided evidence for sex moderation by showing that the mediating pathway from LLS to CRP through BMI, and to a lesser extent through hip/waist circumferences, was significant only in women. In conclusion, perceived LLS was positively associated with the level of inflammation mediated by BMI/hip/waist circumference, with the association between LLS and CRP being stronger in women. These findings contribute to the current literature untangling mediation/moderation processes in which perceived LLS may contribute to adiposity-related inflammation. They also add to precision medicine development, suggesting that stress and inflammation-reducing interventions should focus on African Americans, particularly women.

## 1. Introduction

Ongoing negative life experiences related to an individual’s social roles and personal persistent adverse situations (e.g., long-term financial strain, discriminatory experiences, unfulfilling work situations, and threatening and poor community conditions) operate as chronic life stressors [1,2]. Chronic stressful life experiences over-activate physiological stress response systems (via the hypothalamic-pituitary-adrenal axis and the sympathetic nervous system) resulting in long-lasting physiological dysregulation and pathologic consequences for almost every organ system (endocrine, immune, cardiovascular, nervous), and for body weight and body composition [3,4,5]. Chronic stress also propagates low-grade chronic inflammation, which is well recognized as a major contributor to multiple chronic diseases [5,6,7]. Several serum biomarkers used to evaluate inflammation, such as pro-inflammatory cytokines and C-reactive protein (CRP), are also markers of physiological dysregulation and are used to estimate the risks for various diseases, particularly cardiovascular and metabolic diseases [8]. 

We posit that the challenging (or non-satisfying) life experiences that some middle-aged and older individuals may have endured over the course of their lives can be reflected in their own evaluations of their lived lives [2]. Those who have negative perceptions of their lives may have experienced more life challenges over the course of their lives compared to those who have had fewer challenges [9]. In other words, life satisfaction in the later years may capture the quality of individuals’ cumulative life experiences, resulting in different consequences. Specifically, internalized negative perceptions of each individual’s life (lower life satisfaction) may generate stress and negative emotions [2]. In view of the perpetual relationship between chronic stress and chronic inflammation [5], it is possible that individuals’ lower life satisfaction (LLS) in later years may be associated with chronic inflammation and reflected in elevated levels of some pro-inflammatory markers, including serum CRP, thus subjecting them to higher risk for chronic diseases. 

African Americans are disproportionately exposed to lingering and long-lasting socioeconomic adversities and associated hardships or stressors. These include but are not limited to low income, underemployment, and systematic and day-to-day discrimination. These stressful experiences may start at an early age and accumulate throughout their lives culminating in their later years [1,10,11]. The literature highlights resiliency in African Americans [12,13,14]. For example, in a study using the National Survey of Black Americans between 1980 and 1992, Adams (1997) found that although the situation for African Americans stagnated or declined in health, education, and income during that period, those studied increased general life satisfaction, and there was not a decline in happiness [15]. Becker et.al. even refer to it as a “resiliency philosophy” in chronically ill older African Americans [13]. Finally, Krause (2004) found a link between life satisfaction and religious factors in older African Americans which may explain actual higher life satisfaction than expected in this population [16]. 

However, the association between the life satisfaction and serum CRP may be particularly relevant to this population. Indeed, studies conducted exclusively in African Americans (although rare) showed higher levels of serum CRP in those suffering from everyday discrimination [17], or from discrimination based on socioeconomic status [18]. It is also well established that African Americans, compared to other racial/ethnic groups, experience relatively higher prevalence rates of multiple chronic diseases (e.g., cardiovascular, diabetes, obesity) [19], for which the underlying inflammation may be a cause or have exacerbating consequences. 

Moreover, little is known about the association between life satisfaction and disease risk indicated by elevated CRP levels in middle-aged and older African Americans, especially in a faith-based milieu. An earlier population-based review examined the differences in CRP based on racial/ethnic and socioeconomic determinants and found that, in most cases, poverty and non-white race/ethnicity status were significant contributors to the elevated CRP levels [20]. The unambiguous results were reported in a recent mixed-race study investigating several pro-inflammatory markers (CRP, E-selectin, interleukin-6) in connection with race, perceived discrimination, and socioeconomic status, showing a higher concentration of each marker in African American participants, even after adjusting for common confounders [21]. 

The sex difference in the serum CRP levels seems to be widely agreed upon, showing females having higher levels than males [22,23]. Moreover, studies have documented that higher CRP levels are associated with higher body mass index (BMI) and/or other obesity markers (e.g., body circumferences) [24,25]. Such associations have been attributed to a pathophysiological mechanism of excess adipose tissue (particularly visceral) and its release of pro-inflammatory cytokines that stimulate the liver production and release of CRP [26].

Many studies have documented that various stressful life experiences may adversely affect body weight and obesity [27]. Specifically, Kuroki (2016) showed that LLS and obesity were significantly associated even when socioeconomic factors were controlled for [28]. These studies have also suggested that stressful life experiences influence weight gain by changing the pattern of an individual’s food consumption, promoting energy-dense (“empty calories”) foods and erratic eating patterns, leading to overweight/obesity, prompted by the activation of the sympathetic nervous system and hypothalamic–pituitary–adrenal axis [29]. Moreover, as reviewed recently, chronic stress contributes to the dysregulation of body composition, leading not just to obesity and increased BMI, but also to the deterioration of bone and muscle tissues, possibly resulting in osteosarcopenic adiposity [5,30]. 

The influence of stress on the increase in BMI and other obesity markers and their subsequent influence on CRP levels suggest that body adiposity may operate as a mediating mechanism linking stressful life experiences to CRP [31]. However, the potential mediating influence of body adiposity on the association between lower life satisfaction (as a marker of enduring life experiences of an individual) and CRP levels has not been investigated in middle-aged and older African Americans. Therefore, intragroup analysis using an African American cohort and focusing on individual experiences (as proposed here) may reveal stress-induced physiological processes that are different from the findings based on analyses representing the general population or majority of White individuals as reported in most of the other studies [32]. 

In addition, previous studies have highlighted the importance of considering sex as an effect modifier when analyzing stress–physiological outcome relationships [22], although this moderating effect of sex has been shown to be dependent on the type of stress. For example, and in relation to CRP, Shivpuri et al. (2012) showed that interpersonal stressors are associated with greater elevations in CRP for females than males, while achievement-based stressors are associated with greater elevations in CRP for males than females [22]. However, no study has investigated how sex moderates the relationship between LLS (which may capture stressful life experiences in multiple domains) and physiological outcomes, such as BMI/obesity and CRP, particularly not in middle-aged and older African Americans. Therefore, the cohort studies evaluating the diseases’ risk factors supported by clinical biomarkers may advance our understanding of the mechanisms of disease onset and help in establishing preventive measures and developing new types of therapies [33], all hallmarks of precision medicine. 

**Figure 1:**
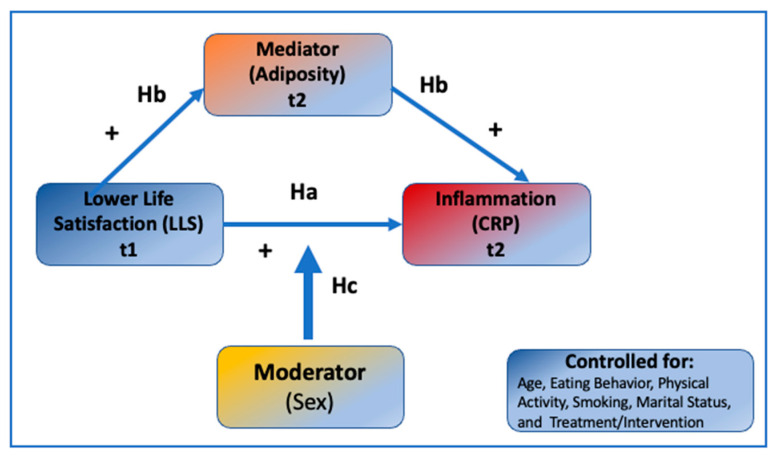
The hypothesized model explaining the relationship between Lower Life Satisfaction (LLS), body adiposity, and inflammation, as reflected in higher C-reactive protein (CRP) serum concentrations.

In view of the above, the present study aimed to enhance our knowledge about the associations between life satisfaction and inflammation in middle-aged and older African Americans, examining how are they mediated by body adiposity (specifically higher BMI and hip and waist circumferences) and moderated by sex, as depicted in Figure 1. Using the data from the cohort of middle-aged and older African Americans collected at baseline and after six months of follow-up, we tested the following hypotheses: (1) (Ha) Perceived poor quality of life (assessed by “Satisfaction with Life Scale” questionnaire) will be positively associated with serum CRP levels, after controlling for age, eating behaviors (expressed as fruit/vegetable/fat intake), physical activity, smoking, marital status, and treatment/intervention; (2) (Hb) this association between perceived life quality and CRP will be mediated by body adiposity (namely, BMI and hip and waist circumferences); (3) (Hc) these hypothesized associations will be moderated by sex.

## 2. Methods

### 2.1. Participants and Inclusion/Exclusion Criteria

Participants were African American adults enrolled in the Reducing Cardiovascular Disease Risk Study that evaluated the effectiveness of a longitudinal church-based intervention, Health for Hearts United [34,35]. The study was conducted in six churches (three treatment, three comparison) in a two-county area of North Florida, with congregants who attended church regularly (at least twice a month). They were randomly sampled in each church and stratified by age and sex (*n* = 221, >45 years), using lists prepared by the church staff and approved by the pastor. There were no special inclusion/exclusion criteria, and all churchgoers meeting age and attendance criteria could participate. The intervention was implemented in 6-month phases representing three conceptual areas: individual knowledge development, clinical learning (individual and small group educational sessions), and efficacy development (recognition and sustainability). In the first six months, the conceptual focus was on individual and his/her knowledge development. The study lasted 24 months with data collected at baseline, 6, 18, and 24 months. The subsample (*n* = 83) with clinical measurements (anthropometries, serum analyses), in addition to all other measurements (see below), was used for this study and assessed at the first and second wave (baseline, t1, and six months, t2). 

All participants (including the subsample) lived in counties in which both the poverty rates and the percentage of African Americans were higher than the state average. Attrition analysis using group mean comparison (t-test) showed that age, marital status, life satisfaction, and perceived physical health of those who participated in the present study (*n* = 83) were not significantly different from the respondents who did not participate in the present study (*n* = 138). 

### 2.2. Measurements

#### 2.2.1. Age and Anthropometries

Participants were classified into eight age categories starting from “45–49” to “more than 91 years”. Anthropometric measurements were performed by trained staff at sessions held at the churches. Height and weight were measured without shoes in indoor clothing to the nearest 0.1 cm and 0.1 kg, respectively, using a Charder stadiometer (Issaquah, WA, USA) and a Tanita digital scale (Arlington Heights, IL, USA), respectively. BMI (kg/m^2^) was calculated. Hip and waist circumferences were measured with non-elastic tape (Issaquah, WA, USA) while the participant exhaled and was standing straight with feet together. The hip was measured over the largest bulge of the buttocks and the waist was measured over the narrowest part of the torso, both to the nearest tenth of cm.

#### 2.2.2. Serum Analyses

Overnight fasting blood samples were collected in red-top tubes (without coagulant); the red blood cells were separated by centrifugation and serum was stored at −80 °C until further analyses. Serum high-sensitivity CRP (hs-CRP) was analyzed in our laboratory using the enzyme-linked immunosorbent assay (ELISA) kits (Enzo Life Sciences, Inc., Farmingdale, NY, USA), according to the manufacturers’ instructions, with 0.01–0.08 mg/L sensitivity ranges. According to the risk categories of serum CRP levels provided by the Centers for Disease Control and Prevention and the American Heart Association [19], individuals with a serum CRP concentration above 3.0 mg/L are considered to be in the high-risk category. 

#### 2.2.3. Lower Life Satisfaction (LLS)

LLS was assessed using four reverse-coded items of the “Satisfaction with Life Scale” questionnaire [9], with examples for questions: ‘‘In most ways, my life is close to ideal’’, ‘‘The conditions of my life are excellent’’, ‘‘So far I have gotten the important things I want in life’’, and ‘‘I am satisfied with my life’’. The 7-point-Likert-type responses (uncoded) ranged from 1 = strongly disagree to 7 = strongly agree (internal consistency ∞ = 0.86), where higher scores indicate lower perceived quality of life, as used previously [2]. 

### 2.3. Treatment Intervention 

The present study used data from the first (baseline, t1) and second (6 months, t2) follow-up collections. The intervention for the longitudinal Reducing CVD Risk Study has been described in detail earlier [34,35]. Briefly, the first six months, representing the individual knowledge development conceptual area, included training for the health leaders on dietary health kick-off events planned and implemented by each health ministry. Since the focus of the present study was on the unique change in inflammation, possibly caused by the lower life satisfaction, the components of this intervention were included solely as control variables (listed below) assessed at baseline.

#### Control Variables

For the partial dietary intake, two questionnaires were used, including a validated single item for fruit and vegetable intake [36] and a single item from the National Cancer Institute (NCI), “Fat Screener” for fat consumption [37]. Habitual physical activity was assessed using the Yale Physical Activity Scale (YPAS) [38]. Smoking and marital status were assessed via the questionnaire using the single items “Do you currently smoke? (1 = yes, 0 = no), and marital status (currently being married = 1, not married = 0), respectively. 

### 2.4. Statistical Analysis 

Structural equation modeling (SEM) with Mplus (Version 8) was used to test the hypothesized model. SEM analysis allowed us to test the hypothesized mediational model in the same analytical framework. The goodness of fit of the model was assessed using the chi-square statistic, comparative fit index (CF ≥ 0.95), and root mean square error of approximation (RMSEA ≥ 0.07). These analyses were controlled for the treatment intervention because approximately half of the respondents participated in the first phase of the intervention. Thus, control variables included age, eating behavior reflecting fruit, vegetable, and fat consumption, physical activity, smoking, and marital status. We used the comparative fit index (CFI ≥ 0.95) and Tucker Lewis Index (TLI ≥ 0.95). To account for the missing data, the Full Information Maximum Likelihood (FIML) procedure was implemented. 

The mediating effects of body adiposity were assessed by examining the indirect effect between the quality of life and CRP through BMI and hip and waist circumferences using the bootstrap procedure in Mplus software [39]. Bias-corrected 95% CIs were computed using 10,000 bootstrapped resamples for an indirect estimate. The moderating effect of sex was tested by comparing models for male and female groups using the chi-square inequality test across two groups. 

## 3. Results

### 3.1. Descriptive Statistics and Zero-Order Correlations

Of *n* = 83 participants, 59% were women. Means (±standard deviations) of age, BMI, CRP, and LLS were 58.6 (9.7) years, 35.0 (8.8) kg/m^2^, 4.7 (6.4) mg/L, and 10.5 (6.7), respectively. Bivariate correlations were mostly in expected directions. LLS was significantly correlated with BMI and CRP (r = 0.33, *p* = 0.0081 and 0.23, *p* = 0.010, respectively). BMI was significantly correlated with CRP (r = 0.31, *p* = 0.009).

The participants’ education attainment ranged from some high school (~10.0%) to Ph.D./M.D./J.D. (2.2%) with a median of some college experience (27.8%). At baseline (baseline, t1), participants reported ages ranging from 43–49 (19%) to over 91 (0.5%) with a median age range of 57–63 years (25.2%). On average, respondents were married (45.0%) with two children (25.4%).

Regarding other control variables in the intervention (age, eating behaviors, physical activity, smoking, marital status, and treatment/intervention), only age, fruit and vegetable consumption, and smoking were correlated with LLS (r = −0.37, r = −0.16, r = −0.25 respectively, *p* < 0.05). Therefore, these bivariate associations among LLS, BMI, and CRP were further explicated in multivariate analyses, as presented below.

### 3.2. Testing Mediational Model

**Figure 2:**
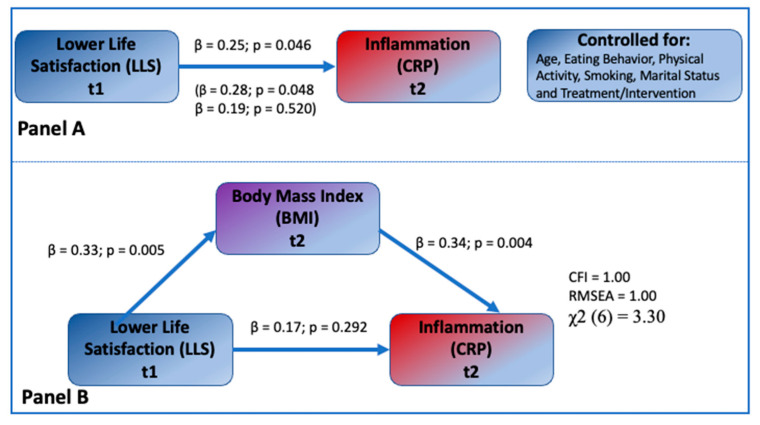
Testing the hypothesized mediational effect of BMI (*n* = 83). Panel A: The direct influence of LLS on CRP. Note: Standardized coefficients are on the top; coefficients for women and men are shown in the parentheses, respectively. Panel B. Mediational Model showing the indirect influence of LLS on CRP through BMI in participants. Note: Standardized regression coefficients are shown. Indirect effect form LLS to CRP through BMI β = 0.11, 95% CI (0.033, 0.263).

Figure 2, Panel A, presents the direct effect of LLS on CRP in all participants (β = 0.25, *p* = 0.046) and separately for females and males, where the direct effects for female and male participants were β = 0.28, *p* = 0.048 and β = 0.19, *p* = 0.520, respectively. Control variables, fruit, vegetable, and fat consumption, physical activity, smoking, marital status, and treatment were not significantly associated with CRP (β’s = 0.14, −0.06, −0.05, 0.25, −0.09, −0.11 respectively, *p* > 0.05 for all coefficients) and not significantly correlated with LLS in the model (not shown in Figure 2). Results from the mediational model showing the indirect effects of LLS on CRP through BMI are shown in Figure 2, Panel B. The associations between LLS and BMI, and BMI and CRP were highly significant (β = 0.33, *p* = 0.005 and β = 0.34, *p* = 0.004, respectively). The indirect effect of LLS on CRP through BMI estimated using the bootstrap procedure was significant with β = 0.11, 95% CI (0.033, 0.263). In this mediational model, the direct association between LLS and CRP was not significant (β = 0.17, *p* = 0.292), suggesting that the previously observed association between LLS and CRP is mediated by BMI. The explained variances of BMI and CRP were 11.1% and 15.2%, respectively. The model showed an acceptable fit with the data (χ2 = 3.30 for 6 degrees of freedom, CFI = 1.00, RMSEA = 1.00). These values remained after including the control variables. 

As a supplementary analysis, we estimated similar models with hip circumference (Hip) and waist circumference (Waist) replacing BMI as the mediator with and without control variables. The results of these mediated models with Hip and Waist without control variables were similar to the results of models with BMI. In these models, both hip and waist circumference mediated the association between LLS and CRP. The mediated paths from LLS to Hip and Hip to CRP were β = 0.32, *p* = 0.009, and β = 0.24, *p* = 0.040, respectively. The mediated paths from LLS to Waist and Waist to CRP were β = 0.29, *p* = 0.010, and β = 0.33, *p* = 0.009, respectively. The direct effects from LLS to CRP were not significant for either Hip or Waist models (β = 0.14, and 0.12, respectively, *p* > 0.05) after accounting for the mediation. The indirect effects were β = 0.16 *, 95% CI (0.053, 0.302) * and β = 0.16 *, 95% CI (0.052, 0.301) *, respectively, for Hip and Waist mediated models, where * indicates *p* < 0.05. 

### 3.3. Testing the Sex Moderation Model

**Figure 3:**
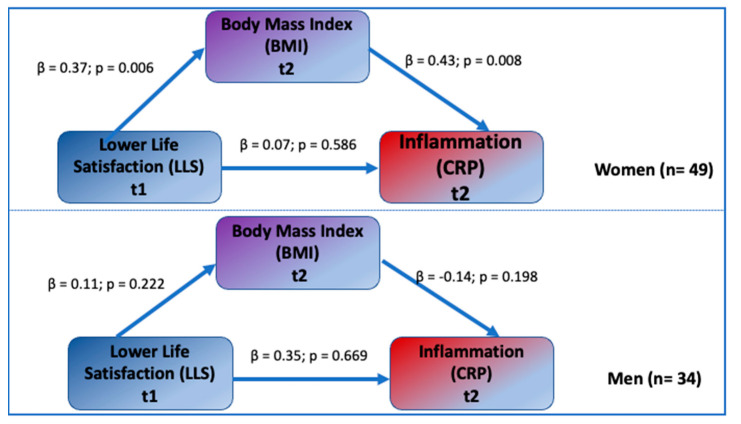
Testing sex moderation. Note: Standardized regression coefficients are shown. The indirect effect from LLS to CRP through BMI β = 0.16 *, 95% CI (0.052, 0.303) for females and β = −0.01 *, 95% CI (0.031, 0.092) for males, where * *p* < 0.05.

Results from the sex moderation model showing the sex difference in the mediational process involving LLS, BMI, and CRP are presented in Figure 3. There was a significant mediational effect of BMI on the association between LLS and CRP in females: the associations between LLS and BMI, and BMI and CRP, were highly significant (β = 0.37, *p* = 0.006 and β = 0.43, *p* = 0.008, respectively). The direct association between LLS and CRP was not significant (β = 0.07, p = 0.586). For females, the indirect effect of LLS on CRP through BMI estimated using the bootstrap procedure was significant (β = 0.16 *, 95% CI (0.072, 0.291) and the explained variances of BMI and CRP were 14% and 22%, respectively. Since the previous results showed that none of the control variables were significantly associated with CRP and since sex group sizes were small, the control variables were not included in these sex-specific models. 

For males, the association between LLS and BMI, and BMI and CRP, were not significant (β = 0.11, *p* = 0.222, and β = −0.14, *p* = 0.198, respectively). The indirect effect of LLS on CRP through BMI was also not significant (β = −0.02, 95% CI (−0.255, 0.213). The direct association between LLS and CRP was not significant (β = 0.35, *p* = 0.669). The explained variances of BMI and CRP were 1% and 15%, respectively.

Chi-square inequality tests showed that the sex difference in the association between BMI and CRP was statistically significant in both female and male groups ∆χ2 (∆df) = 3.89 for 1 degree of freedom, whereas the sex difference in the association between LLS and BMI and CRP did not reach the significant level. 

Similar to the results of the BMI-mediated models, the sex-moderated associations between LLS and CRP were through hip and waist circumferences. The indirect effects (mediations) through hip and waist circumference were significant only for females. For the Hip model, indirect effects for female and male models were β = 0.18 *, 95% CI (0.052, 0.351) and β = −0.07, 95% CI (−0.403, 0.0092), respectively. For the Waist model, indirect effects for female and male models were β = 0.11 *, 95% CI (0.024, 0.251) and β = −0.02, 95% CI (=−0.264, 081), respectively. However, after accounting for the control variables, the effect of Hip/Waist on CRP and the indirect effects through Hip/Waist (mediation) were non-significant in both models. This suggests that hip and waist circumferences are confounded more strongly with control variables than BMI, showing the unique mediating role of BMI in relation to LLS–CRP association.

## 4. Discussion

This study examined the mediational influence of BMI and hip and waist circumferences (as markers of obesity/increased adiposity or its redistribution) influencing the LLS and serum CRP levels (as an inflammatory marker) in middle-aged and older African Americans. Although the direct relationship between LLS and CRP was significant (Figure 2, Panel A), the results of the SEM analysis showed that the association between LLS and CRP was fully mediated by BMI, hip, and waist circumferences, all markers of obesity. In addition, the multi-group SEM analysis provided evidence for sex moderation by showing that the mediating pathway from LLS to CRP through BMI was significant only in women even when controlled for all confounders. 

There are several epidemiological as well as clinical studies in a mixed population showing the positive relationship between elevated serum CRP levels and psychosocial stressors [40,41], including socioeconomic, work, and family stressors. One of the rare studies conducted in African Americans examined everyday discrimination in that group [17], and others examined discrimination based on socioeconomic status in a population of mixed race [18,21]. However, in some studies, these relationships were lost or attenuated when BMI was included as a confounder [17,42,43]. For example, the results of the study conducted in the community-dwelling African Americans (*n* = 296; ~73 years old; 71% women), examining the association of serum CRP and everyday self-reported discrimination, showed a strong positive correlation even when the confounders such as smoking or other existing chronic diseases were included, although these relations lost significance when BMI was included in the analyses [17]. 

Our study, performed in a similar population as that of Lewis et al. [17], including mostly overweight/obese African American women, in a similar socio-economic status (community), corroborates such findings. Based on our results, the loss of significance in the direct relationship between CRP and other psychosocial life adversities when BMI is accounted for could be due to the mediating effect of BMI in these relationships (Figure 2, Panel B). As discussed in the Introduction, chronic stress acts via the hypothalamic–pituitary–adrenal axis and results in pathological endocrine mechanisms leading to adverse body composition outcomes [5,7,29,30], and is most likely involved in these processes. 

Our study is unique based on several aspects. We assessed the mental health component of our participants by measuring their long-life feelings of lower levels of life satisfaction, which reflect the internalized perceptions, experiences, and emotions throughout their entire lives [2,9]. In other words, life satisfaction in the later years, as assessed here, captures the quality of each individual’s cumulative life experiences over the life course. Other studies that evaluated participants’ psychological state of mind by assessing depression, discrimination, or socioeconomic status may not have captured each individual’s cumulative life experiences. Additionally, most of the other studies have been conducted in either White or mixed-race populations [18,24,40,44], and not exclusively in middle-aged and older African Americans, as is the case with our study.

Our previous research showed the connection between LLS and poor diets, physical inactivity, and insufficient sleep in the larger study of middle-aged and older African Americans [2,45], all factors potentially leading to increased obesity and higher risks for metabolic disorders. Results from our other more recent study suggested the persistent unfavorable relationships between LLS, adiposity markers, and CVD risk factors during the longitudinal follow-up [46], prompting us to further investigate the segment of this population. Therefore, the present study extends our previous findings by explaining the relationship between LLS and CRP over time in terms of associated mediation and moderation processes.

Using the SEM modeling enabled us to examine the BMI, hip, and waist circumferences’ mediating process between LLS and CRP within the same analytical framework, providing not only the regression coefficients, but also the overall model fit with the data. Moreover, the SEM model allowed us to test the indirect effect through BMI, hip, and waist circumferences using the bootstrap procedure, which is less sensitive to distributional properties of variables than traditional methods. The SEM model also allowed testing the sex moderation using multi-group comparison, which provided more presentable results. 

Within the sex moderation testing, the SEM analyses showed that the significant mediational effect of BMI, hip, and waist circumferences on the association between LLS and CRP was present only in women. None of the associations between LLS and BMI/hip and waist circumferences, and BMI/hip and waist circumferences and CRP, were significant for men (Figure 2, Panel A, and Figure 3). In contrast, a Friedman et al. study, conducted in a large cohort of Caucasian men and women, showed that both greater lifetime exposure to major discrimination and chronic everyday discrimination (e.g., unfair treatment, job inequality) predicted higher circulating levels of E-selectin (an indicator of endothelial dysfunction and higher cardiovascular risks), but only in men [47]. Our results are also in partial disagreement with Lewis et al., a study in which there was no sex difference in the relationship between CRP and everyday discrimination in older African Americans. Both men and women in that study reported similar discriminatory experiences and the assessed relationship with serum CRP was significant [17]. 

There are several possible explanations why the LLS, BMI, and CRP relationships were not significant in men but were in women, despite their similar (and not significantly different) serum CRP levels and LLS scores. The most likely explanation is based on the endocrine and physiological differences between men and women. For example, we showed recently that African American women compared to men had significantly higher serum leptin levels [48]. This is corroborated with other findings showing higher leptin levels in African Americans in general, but particularly in African American women [49]. A Caffo et al. study showed that serum leptin was positively associated with anthropometric measurements (waist and hip circumferences, and BMI, in multiple regression models), and with serum CRP (in bivariate correlations). In addition, this relationship was strongly influenced by the female sex, indicating possible leptin dysregulation affecting African American women more than men [48]. Hyperleptinemia, preceding or indicating leptin resistance, may create a pro-inflammatory environment, all potentially leading to obesity, diabetes, and increased cardiovascular risks [50]. Therefore, African American women in our study were already in a disadvantageous endocrine status compared to men, which made the relationships found here more salient.

Furthermore, it is well established that body adiposity, especially visceral fat, but also subcutaneous fat, influences inflammatory response, increasing the secretion of some hormones (e.g., leptin, adiponectin) and pro-inflammatory cytokines (e.g., CRP, interleukin-6, E-selectin, tumor necrosis factor) [51]. This response seems to be also sex-specific, as women typically have greater body fat (particularly visceral) compared to men, even when their BMIs are similar. In our study, men had slightly but not significantly higher BMI compared to women, probably due to their overall higher height and weight, the latter reflecting higher muscle mass. However, the waist circumference (an indicator of visceral adiposity and the site of proinflammatory cytokines secretion [51]) was 18.2% above normal in women, compared to 5.7% above normal in men. Similar higher percentages above normal for women than men were noted for the hip circumference. Therefore, women may have been in a higher pro-inflammatory state, to begin with, which was not necessarily reflected only in CRP levels, but also in other cytokines. It is possible that such an overall higher proinflammatory state in women afforded an environment for stronger relationships with other factors examined here and sex moderation. 

Although race discrimination affects the mental health of both men and women, women may also be experiencing sex discrimination [52]. This is another component that could have contributed to more significant LLS, BMI, and CRP relationships in females compared to male participants of our study. In addition, as shown recently [53], African American women were more likely to try to do something about and/or had more severe reactions to overall discrimination, whereas African American men were more likely to accept discrimination as a fact of life. These findings suggest sex differences in behavior concerning both sex and overall discrimination, thus affecting women more severely than men and possibly causing stronger physiological responses in the former.

There are strengths and limitations to our study. A major strength is that it is one of just a few studies that focused on the relationship between LLS and CRP in African American adults with similar demographics, history, and living style, providing for the accumulation of the specific data for precision medicine development. In addition, it is one of the few studies that has examined a mental health component within a longitudinal church-based intervention. Using the SEM analyses enabled us to clarify the roles of BMI hip and waist circumferences, and sex in terms of the association between LLS and CRP.

Life satisfaction was assessed via the self-reported questionnaire, and utilized brief, global scales to decrease the participant’s burden. This might have introduced some errors of a subjective nature, which is typical for such kinds of assessments. However, this questionnaire was previously used in this population and was proven to be a good assessment tool [2,45,46]. The number of participants was relatively small because only those with complete clinical data points were utilized in the analyses. In particular, the number of male participants was lower (*n* = 34) than the female participants (*n* = 49), possibly causing the loss of significance of the sex-moderating power in the former group. However, the underrepresentation of AA males in research studies is a long-going and persistent problem [54]. 

## 5. Conclusions

In conclusion, the results of our study contribute to the clarification of the relationship between lower life satisfaction and inflammation, the latter expressed as CRP in middle-aged and older African Americans, showing that the process is mediated by BMI and, to a lesser extent, by hip and waist circumferences in women. Therefore, our hypothesized objectives were confirmed in this six-month follow-up study: persistent lower life satisfaction, originating from life-long perceived and/or experienced adverse events, predicted higher inflammatory state (particularly in women) which, in turn, may lead to several chronic diseases. 

The implications of these results, pertinent for precision medicine development and in a broader sense, suggest that inflammation-reducing interventions should focus on African Americans, but particularly women as a group facing the highest psychosocial disadvantages. Specific health interventions, including diet, exercise, and other healthy lifestyle behaviors, should be included in this population as potential mediators of health adversity. However, the emphasis on the lifestyle as the primary explanation for race-based health disparities overlooks the race-related adversity rooted in structural and cultural conditions, which accelerate biological decline and, thus, the early onset of illness and death in African Americans. Therefore, such issues need to be addressed at the policy level, but they are beyond the scope of this research study.

## Data Availability

The data are not publicly available as it was not part of the informed consent.
